# Several coumarin derivatives and their Pd(ii) complexes as potential inhibitors of the main protease of SARS-CoV-2, an *in silico* approach†

**DOI:** 10.1039/d0ra07062a

**Published:** 2020-09-23

**Authors:** Dejan A. Milenković, Dušan S. Dimić, Edina H. Avdović, Zoran S. Marković

**Affiliations:** Institute for Information Technologies, Department of Science, University of Kragujevac Jovana Cvijića bb 34000 Kragujevac Serbia zmarkovic@uni.kg.ac.rs; Faculty of Physical Chemistry, University of Belgrade Studentski trg 12-16 11000 Belgrade Serbia; Faculty of Science, University of Kragujevac Radoja Domanovića 12 34000 Kragujevac Serbia

## Abstract

The global pandemic of Severe Acute Respiratory Syndrome Coronavirus 2 (SARS-CoV-2) caused many fatalities among people and significantly influenced the global economy. Since efficient treatment is not available, the computational methods in biology and chemistry are a promising starting point towards adequate medication. Three previously synthesized coumarin derivatives and their Pd(ii) complexes were examined for the binding affinity towards the M^pro^ protein of SARS-CoV-2 by molecular docking and compared to two Food and Drug Administration (FDA) drugs, cinanserin and chloroquine. All of the investigated compounds bind to the active position of the mentioned protein. Coumarin–Pd(ii) complexes showed higher binding affinities compared to the approved drugs. The bindings of the bis(3-(1-((3-chlorophenyl)amino)ethylidene)-chroman-2,4-dione) palladium(ii) complex, its corresponding ligand, and cinanserin to SARS-CoV-2 M^pro^ were further subjected to the molecular dynamics simulations. The binding free energies, computed by MM/PBSA approach were analyzed in detail and the importance of specific interactions outlined. These results showed that the molecules bearing structural similarity to the approved drugs and their complexes have the potential to inhibit the functional activity of SARS-CoV-2 protease and further experimental studies should be undertaken.

## Abstract 2

Coumarin derivatives and their Pd(ii)-complexes have shown a higher binding potential towards SARS-CoV-2 M^pro^ than chloroquine/cinanserin along with lower toxicity.

## Graphical abstract

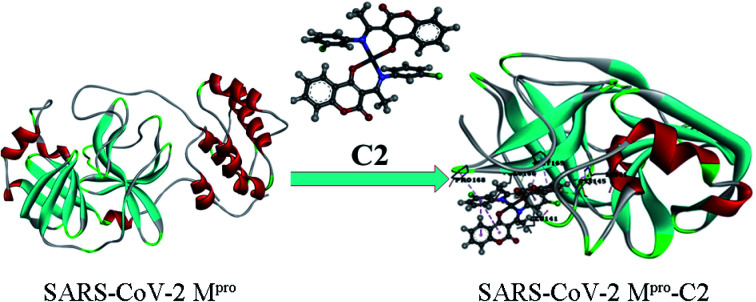

## 1. Introduction

Human Coronaviruses (HCoVs) are pathogens with very long single-strand RNA (30 000 bp), which cause a wide range of diseases through numerous pathogenic mechanisms, and have been found to easily mutate and infect new species.^1,2^ Until December 2019, six human coronaviruses belonging to *Alphacoronaviruses* (HCoV-NL63 and HCoV-229E) and *Betacoronaviruses* (HCoV-OC43, HCoV-HKU1, SARS-CoV, MERS-CoV) were identified.^3^ The most aggressive of these were SARS-CoV (severe acute respiratory syndrome) and MERS-CoV (Middle East respiratory syndrome) human coronavirus, which caused severe, and in most cases, fatal, respiratory infections.^1,2^

The newly discovered coronavirus disease (COVID-19) is an infectious disease caused by the SARS-CoV-2, a human coronavirus from a group of *Betacoronaviruses*. COVID-19 appeared in the City of Wuhan, Hubei Province of China^4^ on 31 December 2019 and the epidemic has spread rapidly in all parts of the world. On September 2, 2020, according to the World Health Organization, this virus has been identified in 213 countries, with 25 935 511 infected, 861 900 dead and 18 217 094 cured. Most infected individuals have mild upper respiratory tract problems and recover without special treatment. Fewer patients, especially those with pre-existing health problems such as diabetes, heart problems, and high blood pressure have more severe respiratory problems.^5^ The most common symptoms of COVID-19 are dry cough, fatigue, fever, sore throat.

The problem with combating these infections is the lack of specific treatment for the new virus.^4,6^ For this reason, many scientists and physicians around the world are working intensively to understand the new virus and the pathophysiology of the disease to discover effective therapeutic agents and vaccines.^7–14^ A group of researchers has discovered spike protein, a key protein used by the virus to invade the human cells.^5,15^ This fact opens many new paths that lead to the discovery of the vaccine. It has also been found that the spike proteins have different forms in different coronaviruses.^16^ On the other hand, the second possible target is a 3-chymotrypsin-like protease, a non-structural protein, and a key enzyme in the life cycle^17^ responsible for replication and transcription.^18^ The first approved drug, favilavir, was announced by the National Medical Product Administration of China in February.^3^ After this, the Food and Drug Administration (FDA) approved other drugs like sofosbuvir and ribavirin. Chloroquine is an agent widely used as an antimalarial drug, but it has been found to exhibit a certain level of anti-HIV activity.^19,20^ Wang *et al.* examined *in vitro* inhibitory activity of chloroquine against SARS-CoV-2, and they found that this compound has also acted against the new virus^21^ although there are certain risks of its use.^22–24^Cinanserin, a drug with antiserotonin activity,^25^ also exhibited significant antiviral potential against the SARS-CoV virus.^26^ These two drugs are included in the present research as model systems, with activity proven both theoretically and experimentally.

**Fig. 1:**
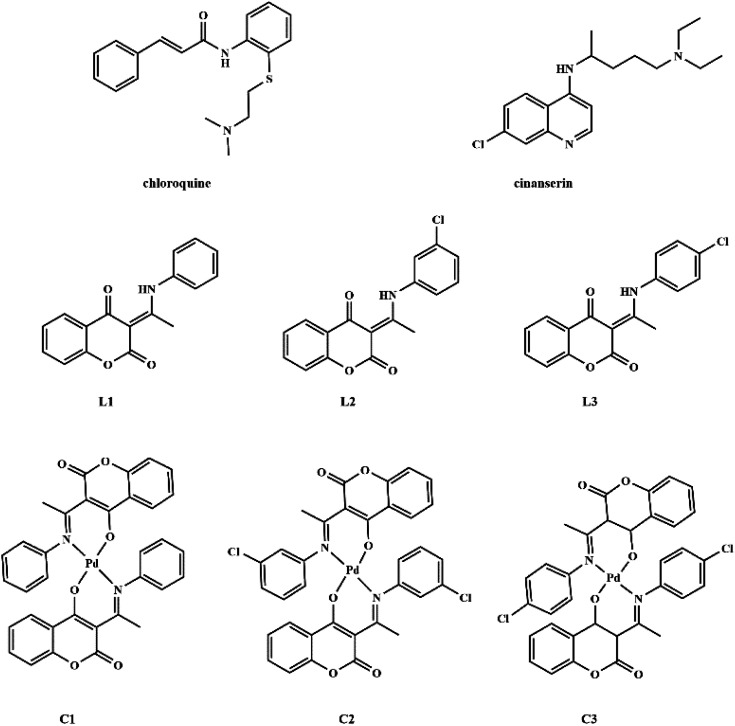
Structures of chloroquine, cinanserin, coumarine derivatives and their palladium(ii) complexes.

Coumarin derivatives are a class of naturally-occurring or modified substances of natural origin that exhibit a broad spectrum of biological functions including antibacterial, antitumor, antioxidant, anti-HIV, and antiviral activity.^27–31^ Due to the similarity in the structure of coumarin derivatives obtained in our group and mentioned drugs, it can be expected that this group of compounds may exhibit activity against SARS-CoV-2 as well. Therefore, the protein binding potential of L1 ((3-(1-(phenylamino)ethylidene)-chroman-2,4-dione)),^32^L2 ((3-(1-((3-chlorophenyl)amino)ethylidene)-chroman-2,4-dione)),^33^ and L3 ((3-(1-((4-chlorophenyl)amino)ethylidene)-chroman-2,4-dione)),^33^ and their palladium(ii) complexes, C1 (bis(3-(1-(phenylamino)ethylidene)-chroman-2,4-dione) palladium(ii) complex),^34^C2 (bis(3-(1-((3-chlorophenyl)amino)ethylidene)-chroman-2,4-dione) palladium(ii) complex),^33^ and C3 (bis(3-(1-((4-chlorophenyl)amino)ethylidene)-chroman-2,4-dione) palladium(ii) complex)^33^ (Fig. 1) towards SARS-CoV-2 main protease is compared to that of antiviral agents of chloroquine and cinanserin, using molecular docking and molecular dynamics (MD) simulations with special emphasis on possible interactions. The physicochemical and pharmacokinetic properties of the title compounds were analyzed by SwissADME, while toxicity was estimated by ProTox II.

## 2. Methodology

### 2.1 Preparation of the SARS-CoV-2 M^pro^ and SARS-CoV M^pro^ structures

The genome sequence of new coronavirus, SARS-CoV-2 (NC_045512.2) was downloaded from the National Center for Biotechnology Information (NCBI) nucleotide database.^35^ The homology model for the main protease (M^pro^) in SARS-CoV-2 was built based on the sequence identity between the SARS-CoV-2 and SARS CoV M^pro^s. The sequence of SARS CoV structure (PDB ID: 2A5I, chain A)^36^ was used as a template that shares 96.08% of the sequence with the SARS-CoV-2 M^pro^ bound to peptide inhibitor N3 (PDB ID: 6LU7).^37^ The Swiss Model webserver was used to build a homology model for M^pro^s by using its automated mode.^38^ The three-dimensional (3D) structure of M^pro^s targeted viruses was used for molecular docking simulations.

### 2.2 Molecular docking

To understand better the binding potential of the coumarin derivatives and FDA approved drugs the molecular docking simulations were employed. Before molecular docking simulations, the pockets and binding sites of M^pro^ of targeted viruses (SARS-CoV and SARS-CoV-2) were determined. For this purpose, the AutoGridFR (AGFR) program^39^ was used. The crystal structures of these proteases were downloaded from RCSB Protein Data Bank in PDB format. The proteins were prepared for docking in Discovery Studio 4.0.^40^ The Kollman partial charges and polar hydrogens were added using the AutoDockTools (ADT) graphical interface. The ligands and their complexes were prepared for docking by optimization of their geometries by density functional theory (DFT) employing global hybrid Generalized Gradient Approximation (GGA) functional B3LYP with empirical dispersion corrections D3BJ (with Becke and Johnson damping)^41^ in combination with the 6-311+G(d,p) basis set for C, N, O, Cl, and H, and def2-TZVPD, triple-zeta-valence, basis set for Pd,^42^ that is implemented in the Gaussian 09 software.^43^ The mentioned functional was used because it describes interatomic interactions at short and medium distances (≤5 Å) more accurately and reliably than traditional DFT methods, and that it reproduces well the crystallographic bond lengths and angles, wavenumbers, and chemical shifts of ((3-(1-(phenylamino)ethylidene)-chroman-2,4-dione)) that is investigated in this article. In the ADT program, the structure of the ligands/complexes was set to be routable, while the protein was kept as a rigid structure. The Lamarckian Genetic Algorithm (LGA) method was used for protein–ligand/complexes flexible docking. For the LGA method, the parameters were determined as follows: a maximum number of energy evaluations was 250 000, a maximum number of generations was 27 000, and mutation and crossover rates were 0.02 and 0.8, respectively. The AutoDock 4.2 software is based on algorithms that can predict the position of compounds within the protein target and to assess them by scoring functions by setting the grid box. The grid boxes with dimensions 52 × 60 × 52 Å^3^ and 64 × 60 × 54 Å^3^ in -*x*, -*y*, and -*z* directions of SARS-CoV-2 M^pro^ and SARS-CoV main peptidase were used to cover the protein binding site and accommodate ligand to move freely. A gridpoint spacing of 0.375 Å was used for auto grid runs. The binding affinity of investigated compounds was examined by the AutoDock 4.2 software.^44^ The binding affinity calculation details are given in ESI.† The three-dimensional (3D) results of the interactions between the target protein and investigated compounds were analyzed and illustrated in Discovery Studio 4.0 and AutoDockTools.

### 2.3 Molecular dynamics

The SARS-CoV-2–C2, SARS-CoV-2–L2, and SARS-CoV-2–cinanserin docked complexes were used as the starting models for the MD simulations. The parameterization of title ligands was performed by the CHARMM36 force,^45^ while the preparation of complex protein–ligand structure inputs for equilibration and the production was done using the CHARMM-GUI web server.^46^ The 0TIP3P solvation model was applied for the solvation of investigated systems, while the sodium chloride ions were added to neutralize the systems to a salt concentration of 0.15 M in KCl. In this way, the neutralized systems were energetically minimized by steepest descent and conjugate gradient algorithms with up to a tolerance of 1000 kJ mol^−1^ nm^−1^ during of 5000 steps. After energy minimization, each system was equilibrated at human body temperature 310.15 K using the Berendsen weak coupling method in *NVT* (constant volume) ensemble condition with a 2 ns time scale. The production MD phase was performed in the *NPT* ensemble using the LINCS algorithm for a 100 ns time scale including a modified Berendsen thermostat (*τ*_T_ = 1 ps) and a Parrinello–Rahman barostat (*τ*_P_ = 2 ps).^47^ The simulation trajectories were propagated to 100 nanoseconds using the GROMACS 5.1.5 package.^48^ The *g_mmpbsa* program in conjunction with the GROMACS program coupled with Adaptive Poisson–Boltzmann Solver (APBS)^49^ was applied to calculate free energies of binding of protein–ligand complexes. The details of this analysis are presented in ESI†.^50^

The most stable conformations obtained by molecular docking simulations of title compounds were subjected further to the analysis of physicochemical and pharmacokinetic properties of the title compounds employing a free online web server SwissADME. For this purpose, the SMILES notations of structures were applied for calculation.^51^ The toxicity properties of ligands were determined according to the ProTox II web server protocol.^52^

## 3. Results and discussion

### 3.1 Amino acid sequence alignment and homology modeling

**Fig. 2:**
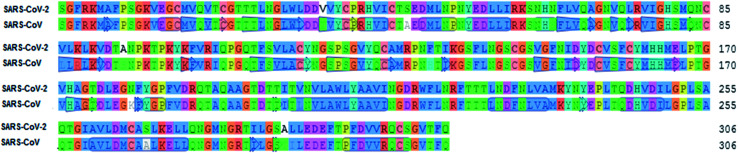
Sequence alignment for the amino acids of M^pro^ between SARS-CoV-2 and SARS-CoV M^pro^.

**Fig. 3:**
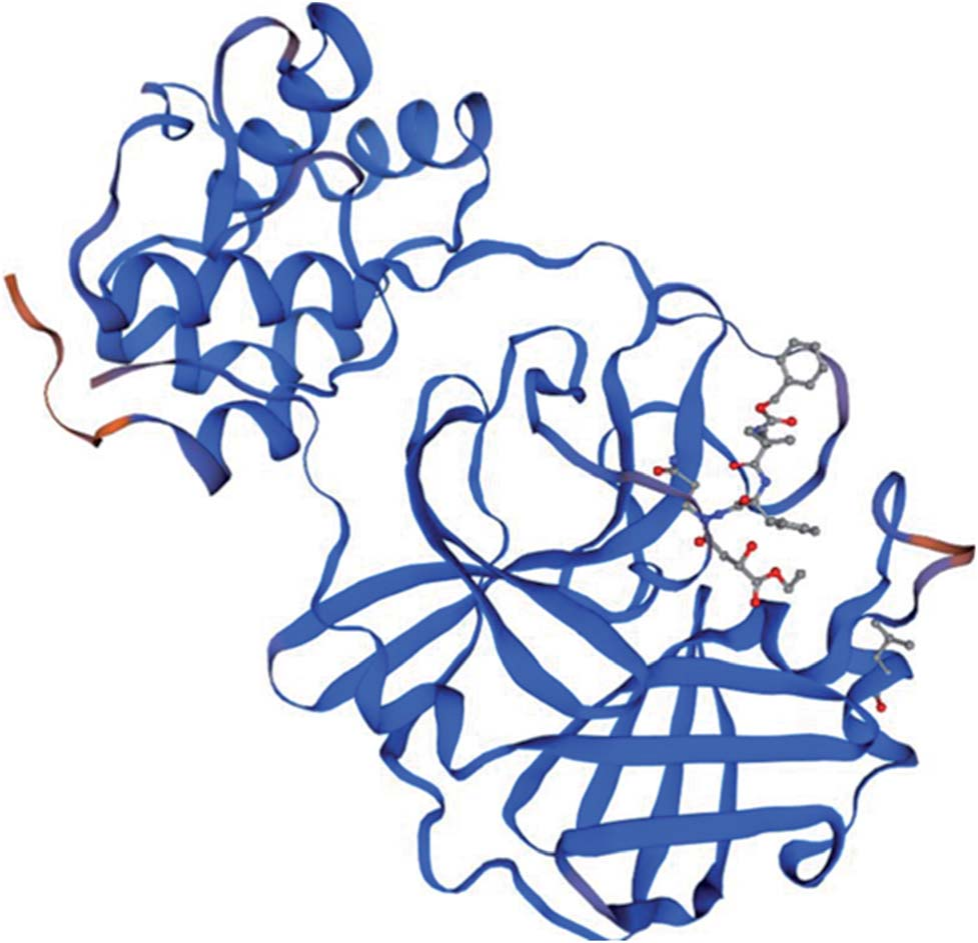
The homology model for the SARS-CoV-2 was built based on the sequence alignment for the amino acids of M^pro^ between SARS-CoV-2 and SARS-CoV M^pro^s. The orange color denotes the difference between these M^pro^s.

To identify specific genomic regions of targeted viruses (SARS-CoV and SARS-CoV-2), the genomic sequences available at the National Center for Biotechnology Information (NCBI) nucleotide database were used and alignments of these viruses were performed (Fig. 2). The SARS-CoV-2 M^pro^ model (306 residues) was generated by homology modeling using the Swiss Model web server. The SARS HCoV M^pro^ (PDB ID : 2A5I, chain A) was employed as a template. The sequence identity between the SARS-CoV-2 and SARS M^pro^s indicated that the SwissModel constructed a valid, high-quality model (Fig. 2, 3 and S1†) for the SARS-CoV-2, with a very high (96.08%) sequence identity to the template. The other parameters for the quality of the model are GMQE = 0.99, QMEAN = −0.30, and local quality estimate = 0.62. The Ramachandran plot was used for the testing of the validity of the SARS-CoV-2 homology model. The results showed that from all of the residues in the allowed regions 96.05% residues were in the most favored region (Fig. S1†).

### 3.2 Active site confirmation and molecular docking analysis

**Fig. 4:**
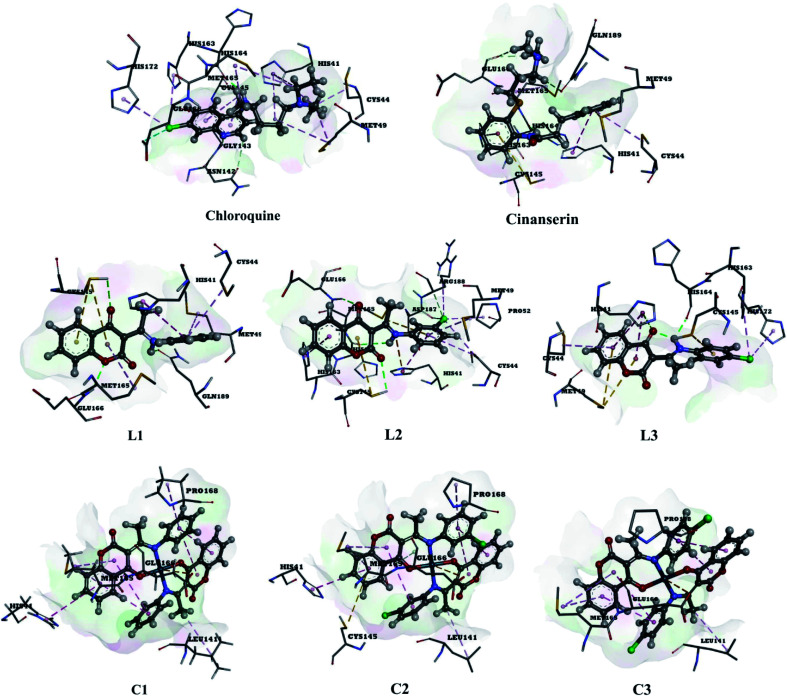
Predicted binding modes between investigated molecules and the active site of SARS-CoV-2 M^pro^.

**Fig. 5:**
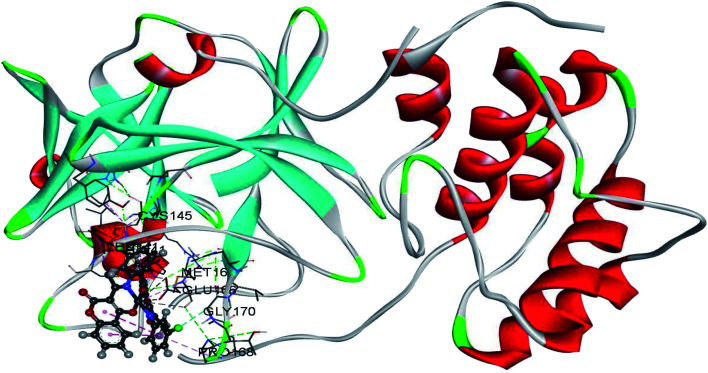
The docking interactions of the most stable conformation of ligand (Pd(ii) complex) with M^pro^ of SARS-CoV-2.

The use of derivatives of previously known antiviral drugs is a useful strategy for the possible accurate treatment methodology for COVID-19. In this study, the protein–ligand interactions of the synthesized coumarin derivatives and FDA drugs with the genome sequence, in the form of a single-chain of RNA, were investigated using the molecular docking simulations. The compounds were chosen in a way that the importance of several structural parameters, such as the presence of halogen atoms, various numbers of aromatic rings, and hydroxyl groups, could be examined along with the transition metal ion of palladium in complexes C1–C3. The structures of investigated compounds, optimized at the B3LYP-D3BJ/6-311+G(d,p) are given in Fig. S2.† In the study by Macchiagodena and coworkers,^53^ it was shown that the molecules with aromatic moieties connected by the rotatable bonds and in the pseudo linear arrangement are promising candidates for the inhibition of investigated protein. Ligands L1–L3 fall within this category. First, the pockets and binding sites of targeted viruses were determined (SARS-CoV and SARS-CoV-2). The AGFR software was employed for this purpose by configuring and computing affinity maps for a receptor molecule to be used for AutoDock4. The bound ligand (N3) was extracted from M^pro^ and binding pocket analysis was performed. After that, re-docking was performed with the synthesized coumarin derivatives and two FDA approved drugs to generate the same docking pose as found in its co-crystallized form. The same protocol was done for the co-crystallized form of SARS-CoV where the aza-peptide epoxide ligand was used. This step was performed to compare the theoretical binding affinity of cinanserin^26^ and correlate it with the experimental inhibition constant. The obtained results of molecular docking studies revealed that SARS-CoV-2 had the Cys–His catalytic dyad (Cys145 and His41) consistent with SARS-CoV (Cys145 and His41) (Fig. 4, Tables S1 and S2†).^36^ These results indicated that the SARS-CoV-2 receptor-binding pocket conformation resembles that of the SARS-CoV binding pocket and raises the possibility that inhibitors intended for SARS-CoV may also inhibit the activity of SARS-CoV-2. The most stable docking conformations of the investigated compound are presented in Fig. 4, 5, Tables S1 and S2.†

#### 3.2.1 Molecular docking analysis of L1–L3 binding to SARS-CoV-2 and SARS-CoV

The results given in Tables 1 and S2† present almost the same trends in binding affinities of investigated molecules towards SARS-CoV-2 and SARS-CoV M^pro^s, with the actual binding free energies values being different for 1–2 kJ mol^−1^. The computed value of the free energy of binding between cinanserin and SARS-CoV is the same as the one presented in ref. 26, which proves that a comparable system is obtained in this study. In the case of SARS-CoV, the free energy of binding for chloroquine has the highest value (−33.8 kJ mol^−1^), followed by L1 (−37.1 kJ mol^−1^), L2 (−37.2 kJ mol^−1^), cinanserin (−37.8 kJ mol^−1^), and L3 (−38.6 kJ mol^−1^). Based on this result, it is expected that L3 would require lower concentrations to inhibit SARS-CoV activity than cinanserin, which showed IC_50_ values of 5 μM *in vitro* and 19–34 μM in tissue cultures studies *in vitro*.^54^ The investigated ligands bind to a receptor M^pro^ of SARS-CoV-2 with higher affinity then chloroquine and cinanserin (Δ*G*_bind_ values are −36.40 and −38.36 kJ mol^−1^, respectively, Table 1), except when L1 is compared to cinanserin. The experimental results have shown that the IC_50_ value for cinanserin inhibition of SARS-CoV-2 M^pro^ is 125 μM.^37^ The higher absolute values of binding energies for ligands L1–L3 for several kJ mol^−1^ than the respective values for FDA approved drugs are expected because the structural features of approved drugs and coumarin derivatives are similar and include two aromatic rings, chorine atom, carbonyl, and amino groups. Coumarin derivatives are further stabilized by the extended delocalization within the structure which prevents the flexibility within the active pocket of protein. Therefore the torsional energies for chloroquine and cinanserin are 10 kJ mol^−1^, while for ligands they are approximately 3 kJ mol^−1^. The presence of chlorine atoms in structure increases the binding affinity when L2 and L3 are compared to L1, which proves the assumption of the importance of this halogen element in the structure.

**Table 1:** The important thermodynamic parameters for best docking conformations of investigated molecules with SARS-CoV-2 (PDB ID: 6LU7)

Conformations	Δ*G*_bind_ (kJ mol^−1^)	*K*_i_ (nM)	Δ*G*_Hbond_ (kJ mol^−1^)	Δ*G*_vdW_ (kJ mol^−1^)	Δ*G*_desolv_ (kJ mol^−1^)	Δ*G*_intermol. energy (vdW+Hbond+desolv)_ (kJ mol^−1^)	Δ*G*_elec_ (kJ mol^−1^)	Δ*G*_total_ (kJ mol^−1^)	Δ*G*_tor_ (kJ mol^−1^)	Δ*G*_unb_ (kJ mol^−1^)	LE
6LU7-chloroquine	−36.40	629.1	−4.47	−45.96	8.17	−42.26	−0.16	−3.97	10.00	0.00	−1.61
6LU7-cinanserin	−38.36	256.8	−2.32	−47.81	7.51	−42.62	−0.25	−5.48	10.00	0.00	−1.57
6LU7-L1	−37.70	338.1	−3.37	−42.85	8.54	−37.68	−0.31	−3.43	3.72	0.00	−1.76
6LU7-L2	−39.95	153.8	−2.21	−46.52	9.99	−38.74	−0.38	−3.35	2.51	0.00	−1.77
6LU7-L3	−39.12	190.6	−2.34	−46.55	9.60	−39.29	0.34	−3.89	3.72	0.00	−1.74
6LU7-C1	−45.13	17.94	−0.20	−47.63	8.79	−39.04	0.18	−8.79	2.51	0.00	−1.03
6LU7-C2	−50.49	2.33	−0.25	−53.86	10.29	−43.81	0.02	−9.20	2.51	0.00	−1.10
6LU7-C3	−47.03	7.55	−0.09	−49.15	9.54	−39.70	0.33	−10.17	2.51	0.00	−1.03

The binding modes of investigated ligands towards SARS-CoV and SARS-CoV-2 M^pro^s are given in Tables S1 and S2.† The catalytically active position around His41 and Cys145 (ref. 26 and 55) is the most active position as all of the investigated molecules bind to this site. The number of interactions explains the difference in binding interactions between ligands and proteins. This number is higher in the case of SARS-CoV-2, which means that ligands are deeper in the binding pocket that is in a more closed state,^56^ and this is well-reflected in values of binding energies. The most prominent interactions are hydrogen bonds, alkyl–π, and π–π interactions (Tables S1 and S2†). The number of hydrogen bonds is important for the overall stability of complexes as suggested by Du and coworkers,^57^ and some of the promising antivirals have over three hydrogen bonds formed in the active center.^55^Cinanserin and chloroquine form one and two hydrogen bonds in the binding pocket of SARS-CoV-2, respectively. The number of hydrogen bonds is higher in the case of ligands L1–3. Glu166 is also important for the stability of protein–ligand complexes with other molecules as hydrogen bonds (L1, L2, and chloroquine) or other non-covalent interactions (L3 and cinanserin) are formed.

#### 3.2.2 Molecular docking analysis of C1–C3 binding to SARS-CoV-2 and SARS-CoV

The results given in Table S2† show that the binding energies for complexes between C1–C3 and SARS-CoV are lower than for other molecules, with the actual values being between −46.0 and −48.2 kJ mol^−1^. On the other hand, the coumarin–Pd(ii) complexes have binding free energies towards SARS-CoV-2 between −45 and −50 kJ mol^−1^. Again, C1–C3 are deeper in the structure of SARS-CoV-2 M^pro^ than SARS-CoV M^pro^. The binding energies of SARS-CoV-2 and investigated compounds complexes are comparable to two approved anti-HIV drugs, indinavir, and darunavir,^56^ but higher than luteolin, ribavirin, remdesivir,^58^ withanone, and caffeic acid.^59^ The stability of these complexes is further increased as strong coordinate bonds are formed between oxygen/nitrogen atoms of amino acids and Pd(ii) ion. The presence of chlorine atom again proved as beneficial because the binding energy of C1 is for 2 and 5 kJ mol^−1^ higher in absolute value than for C2 and C3, respectively. The number of hydrogen bonds is reduced in structures that include Pd(ii) complexes because two active positions, namely NH and the carbonyl oxygen of ligands, are included in the complex formation (Tables S1 and S2†). It is important to notice that the electrostatic interaction between negatively charged Glu166 and positive Pd(ii) ion exists in all docked structures with complexes. Based on the results, it was concluded that the binding of all Pd-complexes to the SARS-CoV-2 M^pro^ is more efficient in comparison to other investigated compounds. C2 with the highest affinity towards receptor M^pro^ of SARS-CoV-2 (Fig. 5) and its corresponding L2 were chosen for further analysis by MD, along with cinanserin which was used for comparison.

### 3.3 Molecular dynamics simulation and analysis

The molecular dynamic analyses were performed with tools in GROMACS Root Mean Square Deviation (RMSD), Root Mean Square Fluctuation (RMSF), and radius of gyration (*R*_g_) to examine the system properties, including the overall stability, local residue, and general structure fluctuations through the simulations. The docked conformations of SARS-CoV-2 M^pro^–C2 and SARS-CoV-2 M^pro^–cinanserin complexes at different simulation intervals are given in Fig. S3.†

The direct changes in the protein from the initial coordinates can be measured by the RMSD (Fig. S4†). These values of the protein backbone in complex with the three potential inhibitors, C2, L2, and cinanserin, were computed for the initial structure as a frame reference (0 to 100 ns). The RMSD values steadily increased from 0 to 5 ns and reached equilibration that remained throughout the simulation period, especially for C2. On the other hand, the RMSD value for cinanserin showed oscillations between 30 to 38 ns indicating that the compound was adapting another confirmation within the binding pocket (Fig. S4†). The average RMSD values for the investigated ligands were 6.82 ± 0.19 (C2 and L2) and 0.32 ± 0.07 Å (cinanserin). It is interesting to note that L2 and C2 have the same value of RMSD which indicates the importance of L2 structure in a molecule for the stability of the formed protein–ligand complex. The difference is expected first because of the size of investigated C2 when compared to cinanserin, and after that due to the formation of stronger bonds with the surrounding amino acids which leads to the change in the protein backbone.

To explore the local protein flexibility, the time average of RMSF values of the 306 amino acids of SARS-CoV-2 protein in the presence of the three possible inhibitors, C2, L2, and cinanserin, over stimulation period was calculated (Fig. S5†). Flexibility is an important property in protein function and more flexible proteins would have enlarged binding pockets which significantly influences the substrate-product kinetics and affinity.^60^ The RMSF values for these complexes suggested that the catalytic dyad residues (His41 and Cys148) showed less fluctuation in all three complexes due to the strong interactions (Fig. S5†). The average RMSF values were the same, 0.17 ± 0.01 nm for all investigated compounds (C2, cinanserin, and L2). The flexibility of protein is reduced in all investigated complexes, but the values do not significantly different due to the interactions with the active positions.

The radius of gyration (*R*_g_) of the protein is associated with its size and compactness. The *R*_g_ values of three complexes were found to be 2.2 nm at the initial state (Fig. S6†). The *R*_g_ value of the complex between a proteins with C2 was stabilized after the initial increase at 5 ns supporting that the systems have reached an equilibrium state. The change in *R*_g_ value for protein–ligand complex with L2 was much more complex, with the decrease until 50 ns and increase until 75 ns. On the other hand, the *R*_g_ value for cinanserin decreased from 10 ns to 20 ns and then it slightly increases up to 40 ns. The latter indicates that the binding of C2 to the protein stabilized its secondary structure (Fig. S6†). The MD simulation results confirmed the stability of all investigated compounds at the active site of SARS-CoV-2.

The obtained docking complexes, SARS-CoV-2 M^pro^–C2, SARS-CoV-2 M^pro^–L2, and SARS-CoV-2 M^pro^–cinanserin, were further subjected to MD energy contribution analysis *via* MM/PBSA protocol,^61^ based on van der Waals, electrostatic, polar solvation, and nonpolar solvation energies (Fig. S7†). The values of various contributions to the total energy are presented in Table 2.

**Table 2:** Important thermodynamic parameters during 100 ns MD simulation generated with MM/PBSA protocol

Complex	Δ*E*_elec_ (kJ mol^−1^)	Δ*E*_vdW_ (kJ mol^−1^)	Δ*G*_polar_ (kJ mol^−1^)	Δ*G*_nonpolar_ (kJ mol^−1^)	Δ*G*_binding_ (kJ mol^−1^)
SARS-CoV-2 M^pro^–C2	−350.2 ± 0.90	−163.4 ± 0.78	386.2 ± 0.79	−22.2 ± 0.05	−149.7 ± 0.63
SARS-CoV-2 M^pro^–L2	−27.4 ± 13.1	−113.8 ± 17.3	91.5 ± 24.2	−12.5 ± 1.7	−62.1 ± 12.8
SARS-CoV-2 M^pro^–cinanserin	−5.8 ± 0.27	−89.7 ± 1.18	57.2 ± 1.09	−11.5 ± 0.2	−49.8 ± 1.04

As listed in Table 2, for SARS-CoV-2 M^pro^, the binding free energy of C2 (−149.7 kJ mol^−1^) is significantly higher in absolute value than that for L2 (−62.1 kJ mol^−1^) and cinanserin (−49.8 kJ mol^−1^). This result indicates the higher binding affinity of the former towards SARS-CoV-2 M^pro^ when compared to the latter. Important to notice is that L2 binds with the higher affinity than cinanserin, therefore both ligand and metal ion contribute to the stability of the SARS-CoV-2 M^pro^–C2 complex. Detailed decomposition of the energy components (Fig. S6†) reveals that the contribution of the energy of electrostatic interactions (Δ*E*_elec_) to the total binding free energy for SARS-CoV-2 M^pro^–C2 (−350.2 kJ mol^−1^) when compared to SARS-CoV-2 M^pro^–L2 (−27.4 kJ mol^−1^) and SARS-CoV-2 M^pro^–cinanserin (−5.8 kJ mol^−1^) is much more prominent. The lower values for SARS-CoV-2–C2 are a consequence of the metal–acceptor bond formation between Pd ion of C2 and oxygen/nitrogen atoms of RdRp amino acid (Glu166) in SARS-CoV-2 M^pro^. The value of Δ*G*_polar_ negatively contributes to the binding process and it is much higher in complex with C2 than in complexes with L2 and cinanserin. The values of nonpolar free energy for SARS-CoV-2 M^pro^–cinanserin (−11.5 kJ mol^−1^), SARS-CoV-2 M^pro^–L2 (−12.5 kJ mol^−1^), and SARS-CoV-2 M^pro^–C2 (−22.2 kJ mol^−1^) complexes slightly contribute to total binding free energy. Based on the obtained results, it can be concluded that the vacuum potential energy (van der Waals and electrostatic interactions) is the major contributor to the total binding free energy and leads to the difference in binding affinities of cinanserin, L2, and C2.

**Fig. 6:**
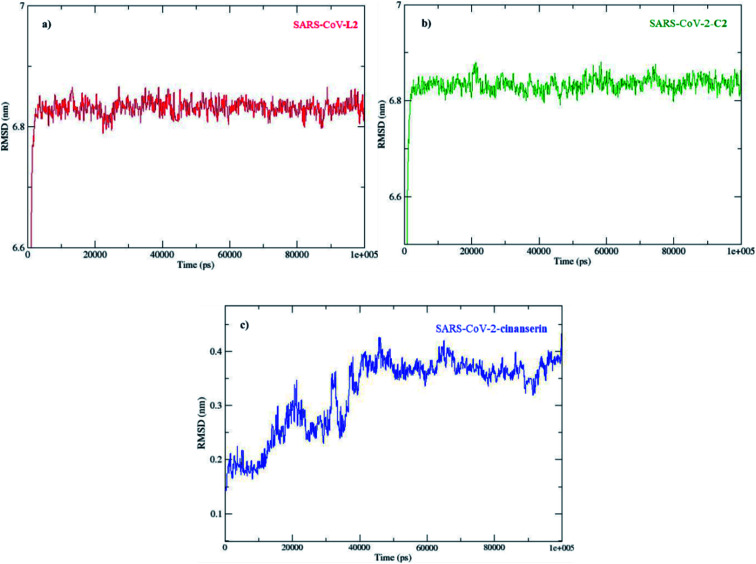
Comparison of the contribution of SARS-CoV-2 residues to the binding free energy in complexes with (a) L2, (b) C2, and (c) cinanserin.

The contribution of the specific residues to the overall binding energy is given in Fig. 6. As seen in Fig. 6b, only the positive contributions are recorded for the complex with C2. The main contribution comes from Glu166, a negatively charged amino acid that interacts with positively charged Pd(ii) ion. Other positive contributions are with His41, Leu141, Cys145, and Met165, which proves that the investigated molecule binds in the active region of SARS-CoV-2 M^pro^ protein. L2 also binds to the mentioned active position of His41 and Cys145 (Fig. 6a). Two methionine residues, namely Met49 and Met165, add significantly to the stability of the complex, while negative contributions are calculated for His163, Glu166, and ASP187. The contribution graph for the SARS-CoV-2 M^pro^–cinanserin complex also shows both positive and negative contributions (Fig. 6c). The amino acids that lead to a positive contribution are Met49, Cys145, and His164. The only amino acid that is the same as for the complex with C2 is Cys145, an important one for the catalytic activity. The negative contribution is recorded for interactions with His41, His163, and Glu166. These interactions may be the reason for the lower affinity of cinanserin towards SARS-CoV-2 than C2 and L2.

### 3.4 Druglikeness and toxicity analysis

The SwissADME web tool was used to compute physiochemical properties and the Absorption, Distribution, Metabolism, and Excretion (ADME) parameters. From this computation, the drug-likeness was predicted. The obtained results are presented in Table S1.† After evaluation of MW (molecular weight), log *P* (predictable permeability of the skin), HB donor (estimated number of hydrogen bonds that would be donated by the solute to water molecules in aqueous solution), HB acceptor (estimated number of hydrogen bonds that would be accepted by solute molecules of water in aqueous solution); according to the rule of five, it was concluded that all compounds satisfy these conditions.^56,57^ Lipinski rules are important in screening methods for new compounds that could inhibit the activity of SARS-CoV-2.^54^ The toxicity was determined by using Prediction of Toxicity of Chemicals (ProTox-II), a virtual web tool lab for predicting the toxicity nature of designed ligands (Table S4†). The toxicity risk assessment such as LD_50_ values in mg kg^−1^ and toxicity class using the PROTOX-II online server was obtained.^46,58^ The oral toxicity values defined for the molecules were 3200 for C2 and C3 and 2647 (mg kg^−1^) for L2 and L3, classifying them as class V, which means that they may be harmful if swallowed (2000 < LD_50_ ≤ 5000). The molecules L1, C1, and cinanserin with LD_50_ values of 1600, 1210, and 480 (mg kg^−1^), are classified in class IV (harmful if swallowed (300 < LD_50_ ≤ 2000)). Class III (50 < LD_50_ ≤ 300) molecules are considered to be toxic if swallowed, and chloroquine with an LD_50_ value of 311 falls within this group (Table S3†). These results lead to the conclusion that the investigated compounds might be less toxic to organisms than approved drugs, although experimental *in vitro*/*in vivo* studies are necessary for the conclusion. Based on these values and results of molecular docking/dynamics it is clear that C2 is a potentially worth candidate for the efficient fight against SARS-CoV-2 and that further experimental examination is required.

## 4. Conclusion

In the present study the binding affinity of three coumarin ligands, namely L1 ((3-(1-((3-chlorophenyl)amino)ethylidene)-chroman-2,4-dione)), L2 ((3-(1-((4-chlorophenyl)amino)ethyl-idene)-chroman-2,4-dione)), and L3 ((3-(1-(phenylamino)ethylidene)-chroman-2,4-dione)) and their corresponding palladium(ii) complexes towards SARS-CoV-2 protease M^pro^ was investigated. The results of the molecular docking study showed that binding affinities of L2, L3, and all of the complexes were higher than those for chloroquine and cinanserin, two FDA approved drugs. All of the molecules bound in the active position of protein, in the vicinity of the His41–Cys145 catalytic dyad. The importance of the presence of halide atom, routable aromatic moiety, and delocalization within the structure was responsible for the high binding affinities. The most stable complexes with proteins, namely L2, its corresponding palladium complex, and cinanserin were subjected to 100 ns molecular dynamic study. The binding free energy of C2 is significantly higher than that for L2 and cinanserin, with values of −149.7, −62.1, and −49.8 kJ mol^−1^, respectively. The toxicity predictions also proved that C2 and L2 belong to toxicity class V, which means that they may be harmful if swallowed, while FDA approved drugs were much more toxic. The newly synthesized molecules also satisfied all of the Lipinski rules. Based on these results, the experimental trials should be undertaken to verify the predictions, but the coumarin derivatives and their transition metal complexes certainly possess significant potential in the global COVID-19 pandemics.

## Conflicts of interest

There are no conflicts to declare.

## Supplementary Material

RA-010-D0RA07062A-s001
