# Supplementary material for: Several coumarin derivatives and their Pd(ii) complexes as potential inhibitors of the main protease of SARS-CoV-2, an in silico approach
Source: RSC Adv. 2020 Sep 23;10(58):35099–108. doi: 10.1039/d0ra07062a (PMC9056878; doi:10.1039/d0ra07062a)
Supplement: RA-010-D0RA07062A-s001 [file RA-010-D0RA07062A-s001.pdf]

Electronic Supplementary Material (ESI) for RSC Advances.
This journal is © The Royal Society of Chemistry 2020

**Supporting Information for:**

# Several coumarin derivatives and their Pd(II) complexes as potential inhibitors of SARS-CoV-2 main protease, *in silico* approach

*Dejan A. Milenković [a], Dušan S. Dimić [b], Edina H. Avdović[a, c], Zoran S. Marković[a*]*

[*] Corresponding author: Prof. Zoran Marković, E-mail: zmarkovic@uni.kg.ac.rs

[a]Institute for Information Technologies, Department of Science, University of Kragujevac, Jovana Cvijića bb, 3400 Kragujevac, Serbia

[b]University of Belgrade, Faculty of Physical Chemistry, Studentski trg 12-16, 11000 Belgrade, Serbia

[c]University of Kragujevac, Faculty of Science, Radoja Domanovića 12, 34000 Kragujevac, Serbia

Methodology

**Molecular Docking – energy contribution**

The AutoDock program calculates these values according to the following equation, Eqn 1:

$$\Delta\mathbf{G}_{bind} = \Delta\mathbf{G}_{vdw+hbond+desolv} + \Delta\mathbf{G}_{elec} + \Delta\mathbf{G}_{total} + \Delta\mathbf{G}_{tor} - \Delta\mathbf{G}_{unb} \quad \text{(S1)}$$

where $\Delta\mathbf{G}_{bind}$ is the estimated free energy of binding, the $\Delta\mathbf{G}_{vdw+hbond+desolv}$ represents the sum of the energies of dispersion and repulsion ($\Delta\mathbf{G}_{vdw}$), hydrogen bond ($\Delta\mathbf{G}_{hbond}$) and desolvation ($\Delta\mathbf{G}_{\mathbf{desolv}}$). The $\Delta\mathbf{G}_{total}$ represents the final total internal energy, the $\Delta\mathbf{G}_{tor}$ is torsional free energy, $\Delta\mathbf{G}_{unb}$ is unbound system's energy, and $\Delta\mathbf{G}_{elec}$ is electrostatic energy. Ligand efficiency (LE) represents the binding energy of ligand to protein per atom. LE (Eqn 2) has a unit of kJ/mol/heavy atom.

$$LE = \frac{\Delta\mathbf{G}_{bind}}{N} \quad \text{(S2)}$$

where N is the number of non-hydrogen atoms.

**Molecular Dynamics – energy contribution**

The free energy of the binding of investigated complex was determined on the following equations [1,2] (Eqn. 3**).**

$$\Delta \mathbf{G}_{bind} = \Delta \mathbf{G}_{complex} - (\Delta \mathbf{G}_{protein} + \Delta \mathbf{G}_{ligand}) \quad \text{(S3)}$$

where $\Delta \mathbf{G}_{bind}$ is the binding free energy of SARS-CoV-2 with **C2** and **cinanserin.** $\Delta \mathbf{G}_{complex}$ is the total free energy of SARS-CoV-2 with investigated compounds, while $\Delta \mathbf{G}_{protein}$ and $\Delta \mathbf{G}_{ligand}$ are total free energies of SARS-CoV-2 protein (PDB ID: 6LU7) and investigated ligands in a solvent, respectively. The free energy of individual molecule was estimated according to the following Eqn. 4.:

$$\Delta \mathbf{G}_{molecule} = \Delta \mathbf{E}_{MM} - TS + \Delta \mathbf{G}_{solvatation} \quad \text{(S4)}$$

where, $\Delta \mathbf{G}_{molecule}$ is the protein or ligand or protein-ligand complex, and $\mathbf{E}_{MM}$ is the average molecular mechanics (MM) potential energy in the vacuum. TS denotes the entropic contribution to the free energy, while T and S represent the temperature and entropy, respectively. On the other hand, $\Delta \mathbf{G}_{solvatation}$ represents the free energy solvation to transfer a solute from a vacuum to the solvent. $\Delta \mathbf{E}_{MM}$ (Eqn. 5) was expressed as:

$$\Delta \mathbf{E}_{MM} = \Delta \mathbf{E}_{bonded} + \Delta \mathbf{E}_{nonbonded} = \Delta \mathbf{E}_{bonded} + \Delta \mathbf{E}_{vdw} + \Delta \mathbf{E}_{elec} \quad \text{(S5)}$$

The $\Delta \mathbf{E}_{bonded}$ denotes the bonded interactions are contributing the bond, angle, dihedral and improper interactions. $\Delta \mathbf{E}_{nonbonded}$ is the nonbonded interaction involving the van der Waals electrostatic energy terms. $\Delta \mathbf{E}_{bonded}$ is always taken as zero. $\Delta \mathbf{G}_{solvatation}$ is an energy term calculated in an implicit solvent and was characterized in Eqn. 6 as:

$$\Delta \mathbf{G}_{solvatation} = \Delta \mathbf{G}_{polar} + \Delta \mathbf{G}_{nonpolar} \quad \text{(S6)}$$

The $\Delta\mathbf{G}_{polar}$ refers to solvation free energy of electrostatic interactions calculated by solving Poisson-Boltzmann (PB) equation, and $\Delta\mathbf{G}_{nonpolar}$ represents to non-electrostatic interactions to the solvation free energy which refers the solvent-accessible surface area (SASA) model. The obtained results of the average binding energy (kJ $mol^{-1}$) calculations can offer a better insight into the interactions of the above-mentioned complexes.

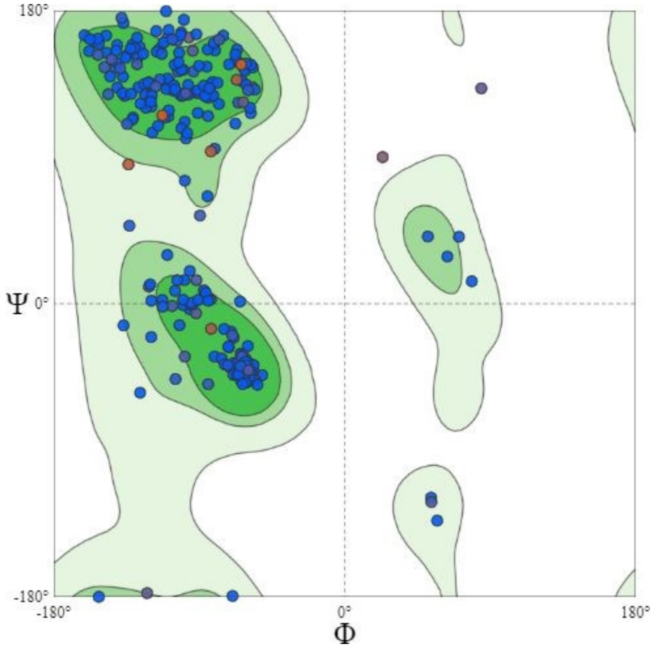


**Figure S1**. The Ramachandran plot of SARS-CoV-2 homology model.

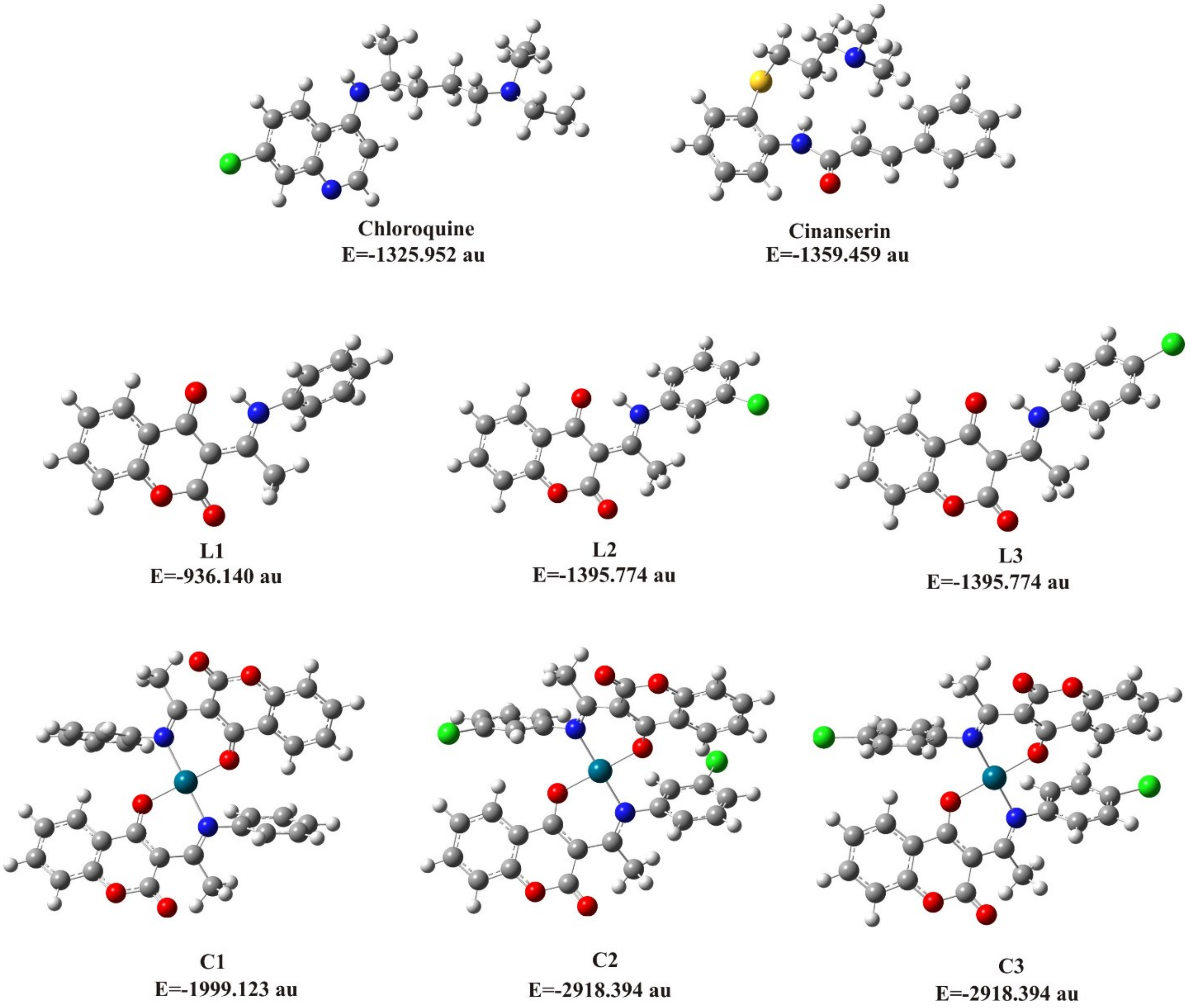


**Figure S2.** Optimized geometries of the ligands and their palladium (II) complexes obtained at the B3LYP-D3BJ/6–311+G(d,p) level of theory, along with the minimal energies.

**Table S1.** Druglikeness of potential inhibitor candidates and interaction with SARS-CoV-2 (6LU7).

| Compound | Protein-ligand interactions | Lipinski's rule of five | |
|---|---|---|---|
| | | **Properties** | **Value** |
| **Chloroquine** | 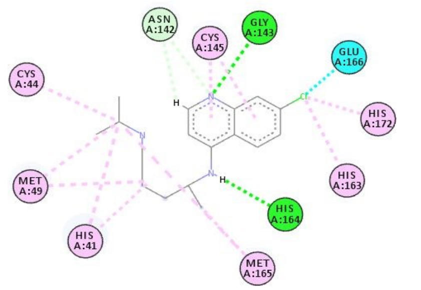 | Molecular weight (<500 Da) | 319.9 |
| | | LogP (<5) | 4.2 |
| | | H-Bond donor (5) | 1 |
| | | H-bond acceptor (<10) | 2 |
| | | Violation | 0 |
| | | Meet RO5 criteria | YES |
| **Cinanserin** | 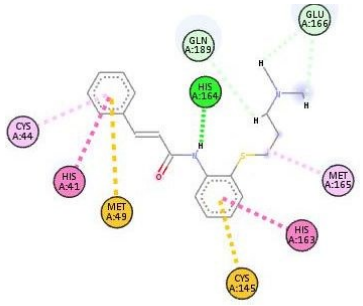 | Molecular weight (<500 Da) | 340.5 |
| | | LogP (<5) | 3.9 |
| | | H-Bond donor (5) | 1 |
| | | H-bond acceptor (<10) | 2 |
| | | Violation | 0 |
| | | Meet RO5 criteria | YES |
| **L1** | 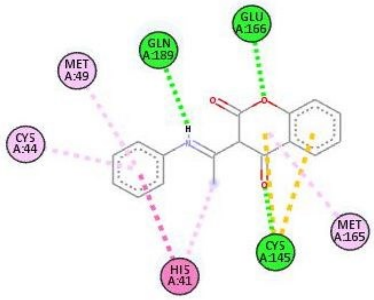 | Molecular weight (<500 Da) | 280.3 |
| | | LogP (<5) | 1.7 |
| | | H-Bond donor (5) | 1 |
| | | H-bond acceptor (<10) | 3 |
| | | Violation | 0 |
| | | Meet RO5 criteria | YES |
| **L2** | 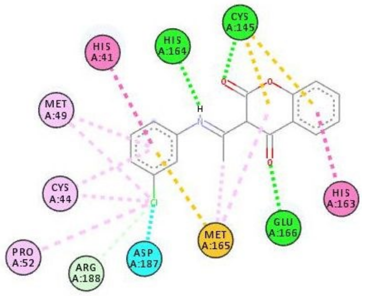 | Molecular weight (<500 Da) | 314.7 |
| | | LogP (<5) | 2.1 |
| | | H-Bond donor (5) | 1 |
| | | H-bond acceptor (<10) | 3 |
| | | Violation | 0 |
| | | Meet RO5 criteria | YES |
| **L3** | 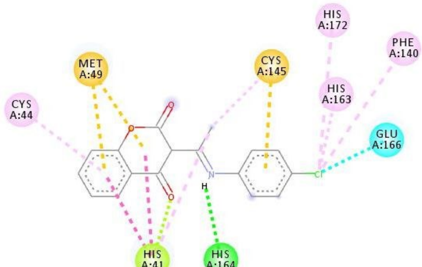 | Molecular weight (<500 Da) | 314.7 |

| | | | |
|---|---|---|---|
| | | LogP (<5) | 2.1 |
| | | H-Bond donor (5) | 1 |
| | | H-bond acceptor (<10) | 3 |
| | | Violation | 0 |
| | | Meet RO5 criteria | YES |
| **C1** | 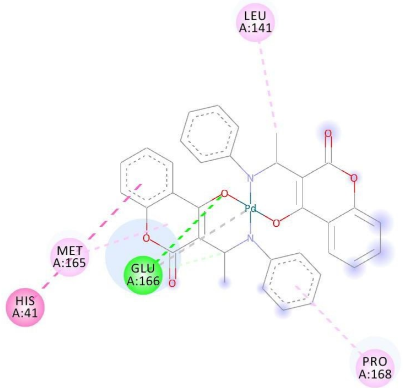  | Molecular weight (<500 Da) | 665.0 |
| | | LogP (<5) | 4.2 |
| | | H-Bond donor (5) | 0 |
| | | H-bond acceptor (<10) | 6 |
| | | Violation | 1 |
| | | Meet RO5 criteria | YES |
| **C2** | 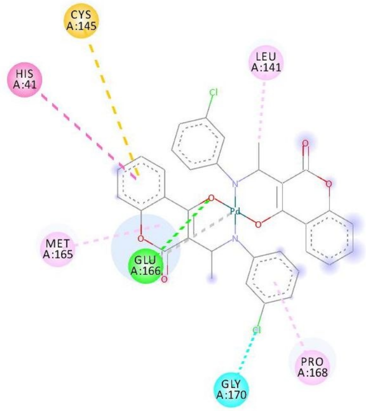  | Molecular weight (<500 Da) | 733.9 |
| | | LogP (<5) | 4.9 |
| | | H-Bond donor (5) | 0 |
| | | H-bond acceptor (<10) | 6 |
| | | Violation | 2 |
| | | Meet RO5 criteria | YES |
| **C3** | 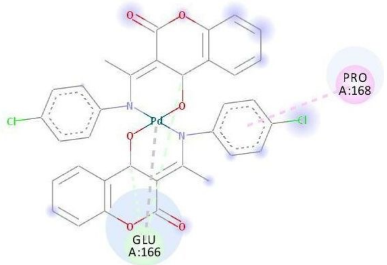  | Molecular weight (<500 Da) | 733.9 |
| | | LogP (<5) | 4.9 |
| | | H-Bond donor (5) | 0 |
| | | H-bond acceptor (<10) | 6 |
| | | Violation | 2 |
| | | Meet RO5 criteria | YES |

**Table S2.** Protein-ligand interactions of the investigated compound with SARS-CoV (2A5I).

| Compound | Protein-ligand interaction | Binding Energy ($\Delta G^{\#}$, kJ/mol) |
|---|---|---|
| **Chloroquine** | 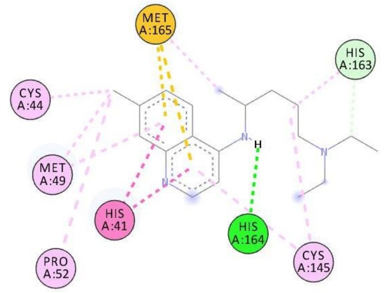 | -33.8 |
| **Cinanserin** | 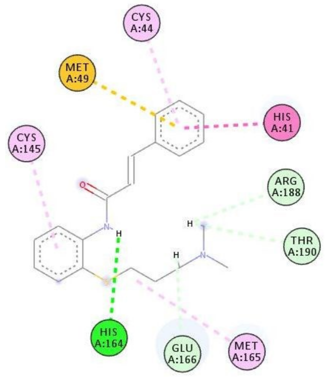 | -37.8 |
| **L1** | 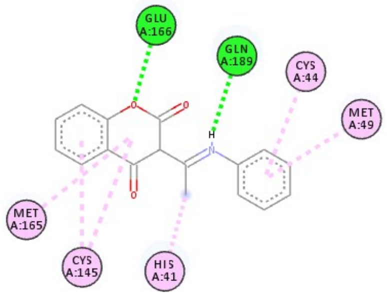 | -37.1 |
| **L2** | 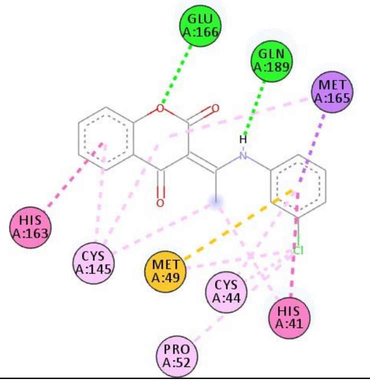 | -37.2 |

| | | |
|---|---|---|
| **L3** | 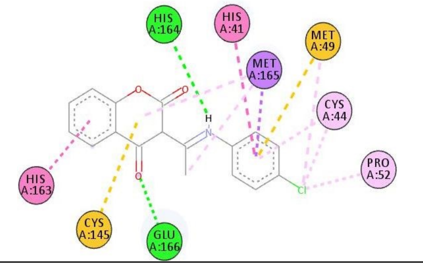 | -38.6 |
| **C1** | 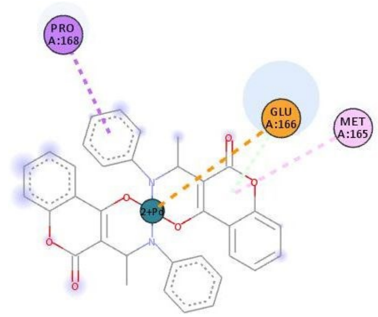 | -46.0 |
| **C2** | 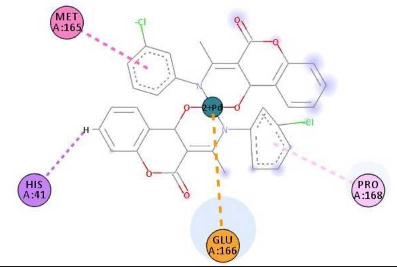 | -48.2 |
| **C3** | 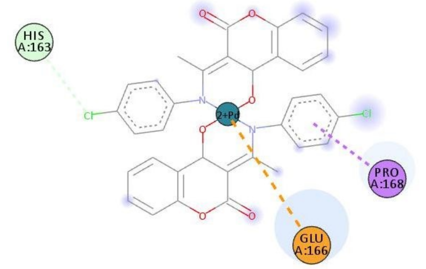 | -46.1 |

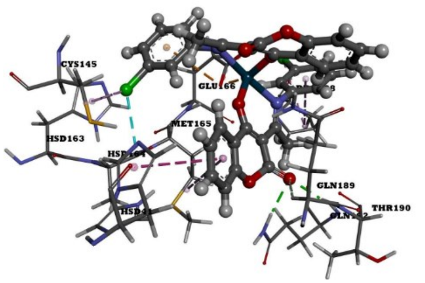

**25 ns of $M^{pro}$-C2**

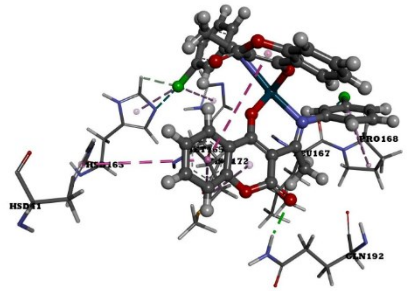

**50 ns of $M^{pro}$-C2**

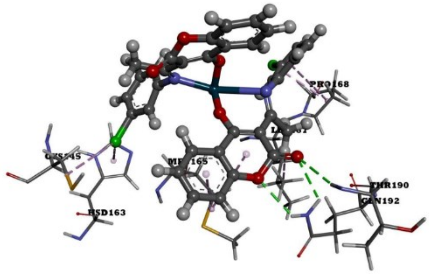

**75 ns of $M^{pro}$-C2**

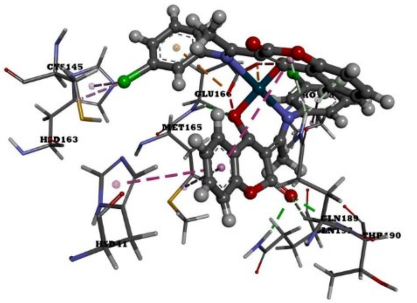

**100 ns of $M^{pro}$-C2**

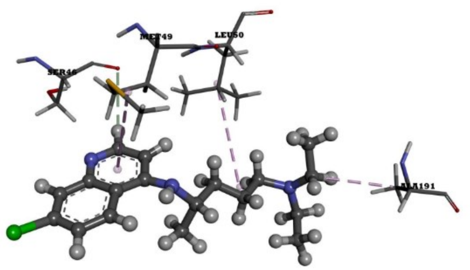

**25 ns of $M^{pro}$-cinanserin**

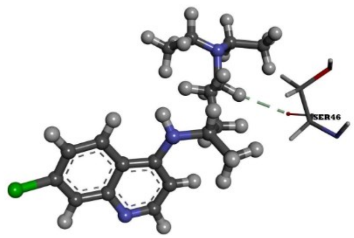

**50 ns of $M^{pro}$-cinanserin**

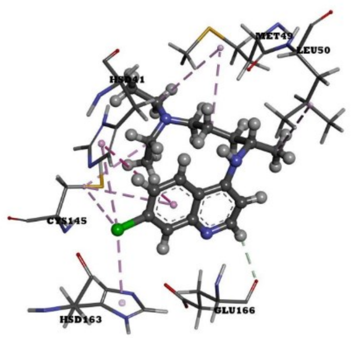

**75 ns of $M^{pro}$-cinanserin**

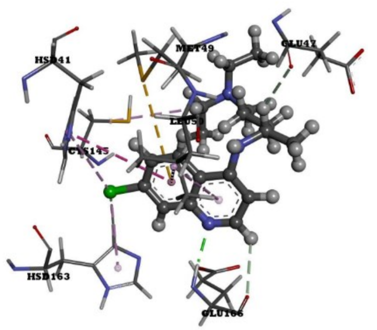

**100 ns of $M^{pro}$-cinanserin**

**Figure S3.** Docked conformations at different simulation intervals for SARS-CoV-2 Mpro-C2 (first row) and SARS-CoV-2 Mpro-cinanserin (second row)

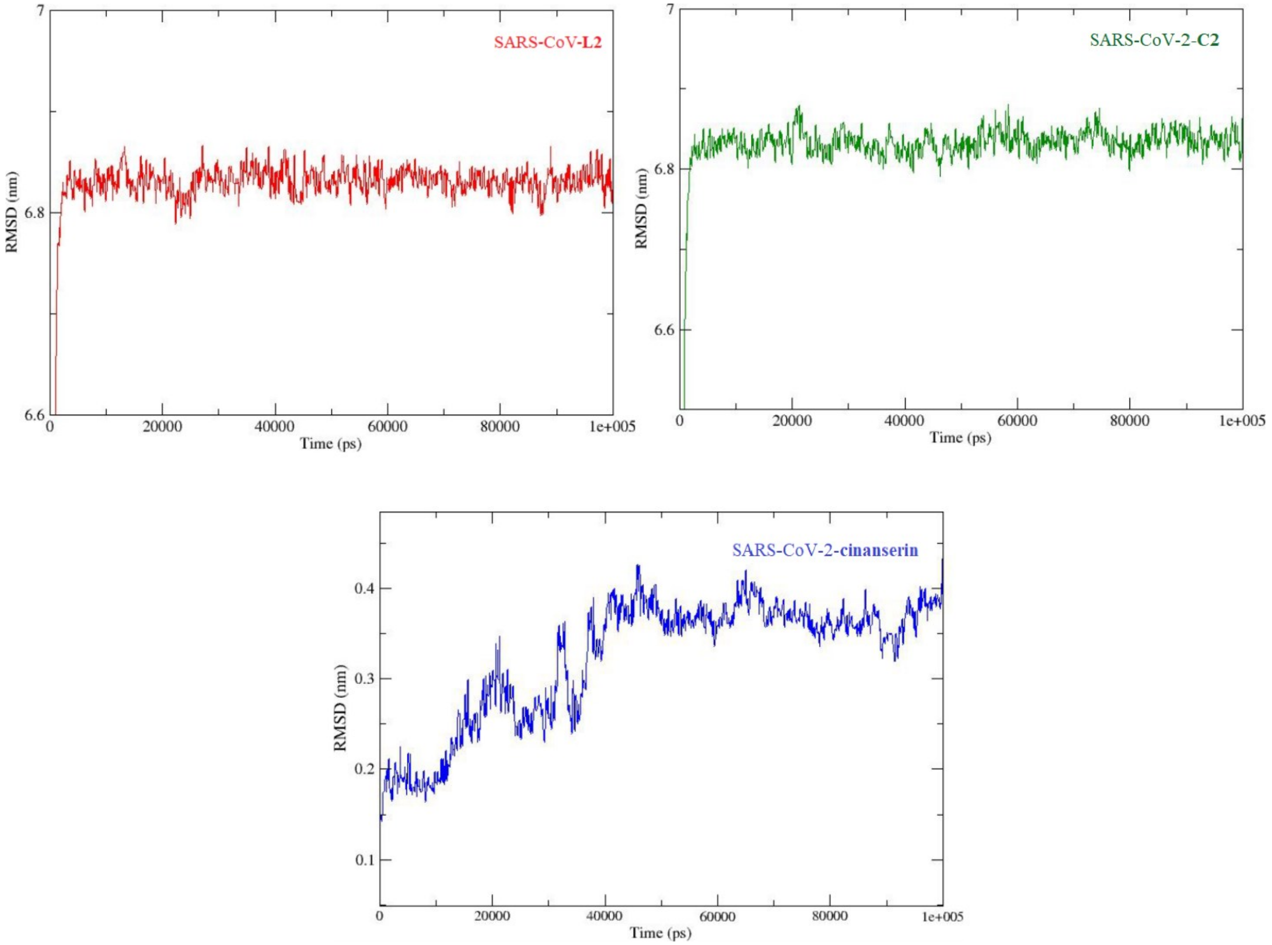


**Figure S4**. Plot of root mean square deviation (RMSD) of C–Cα–N backbone vs. simulation time for solvated SARS-CoV-2 protease in complex with the three candidate compounds during 100 ns molecular dynamics simulations.

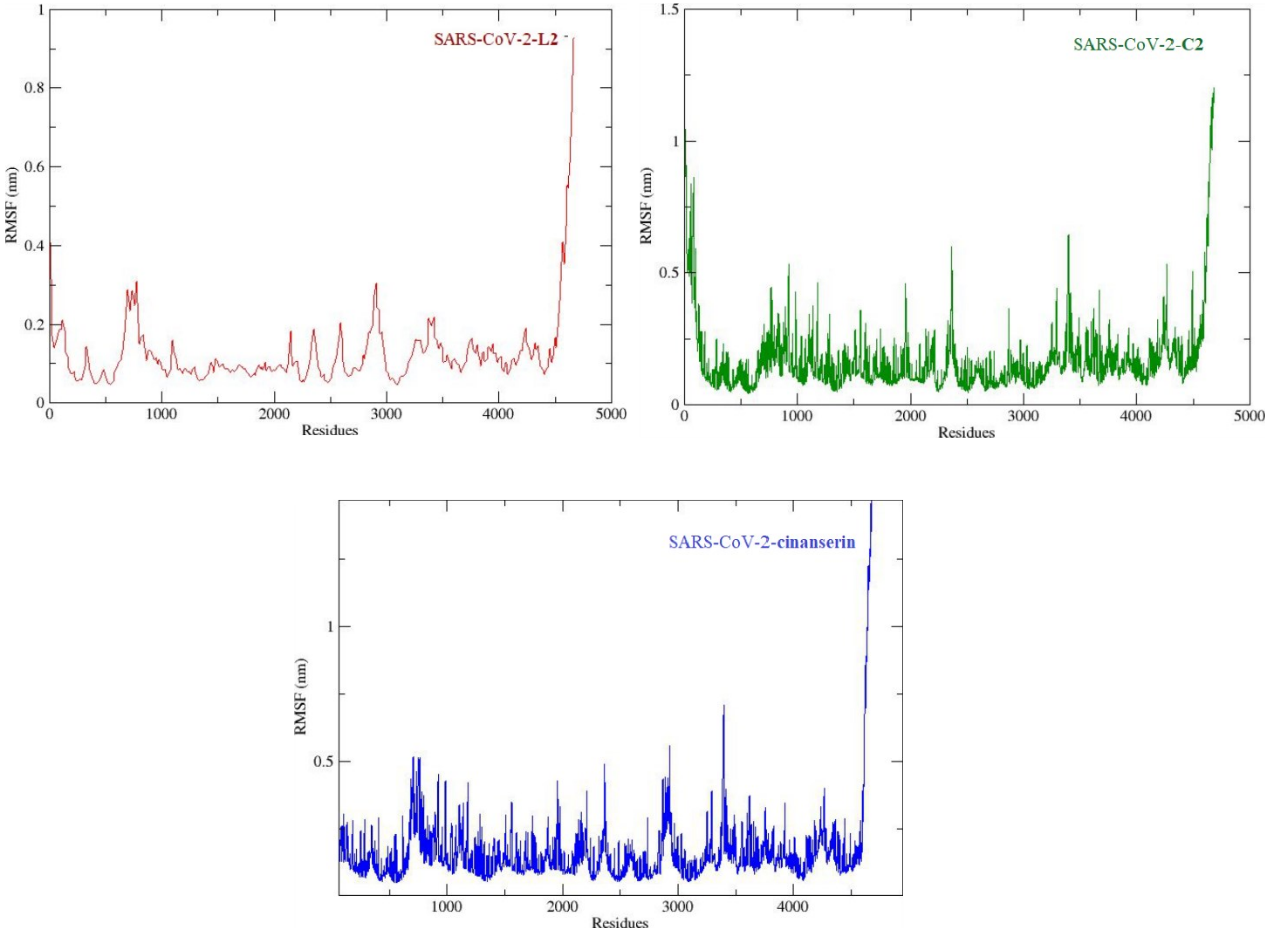


**Figure S5:** The root mean square fluctuation (RMSF) values of SARS-CoV-2 protease in complex with the investigated candidate compounds were plotted against residue numbers.

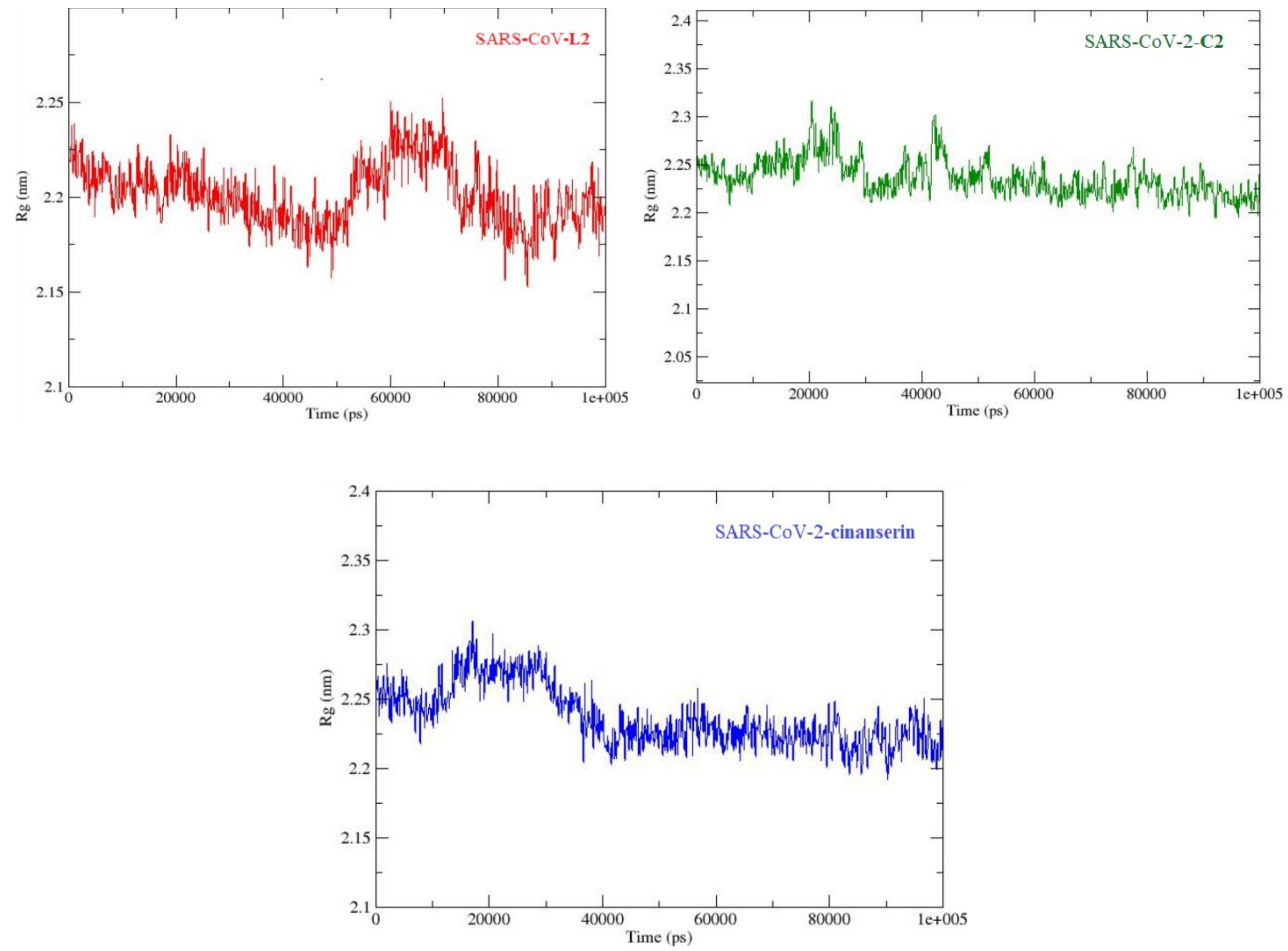


**Figure S6:** Plot of radius of gyration (Rg) during 100 ns MD simulation of SARS-CoV-2 protease in complex with the two candidate compounds.

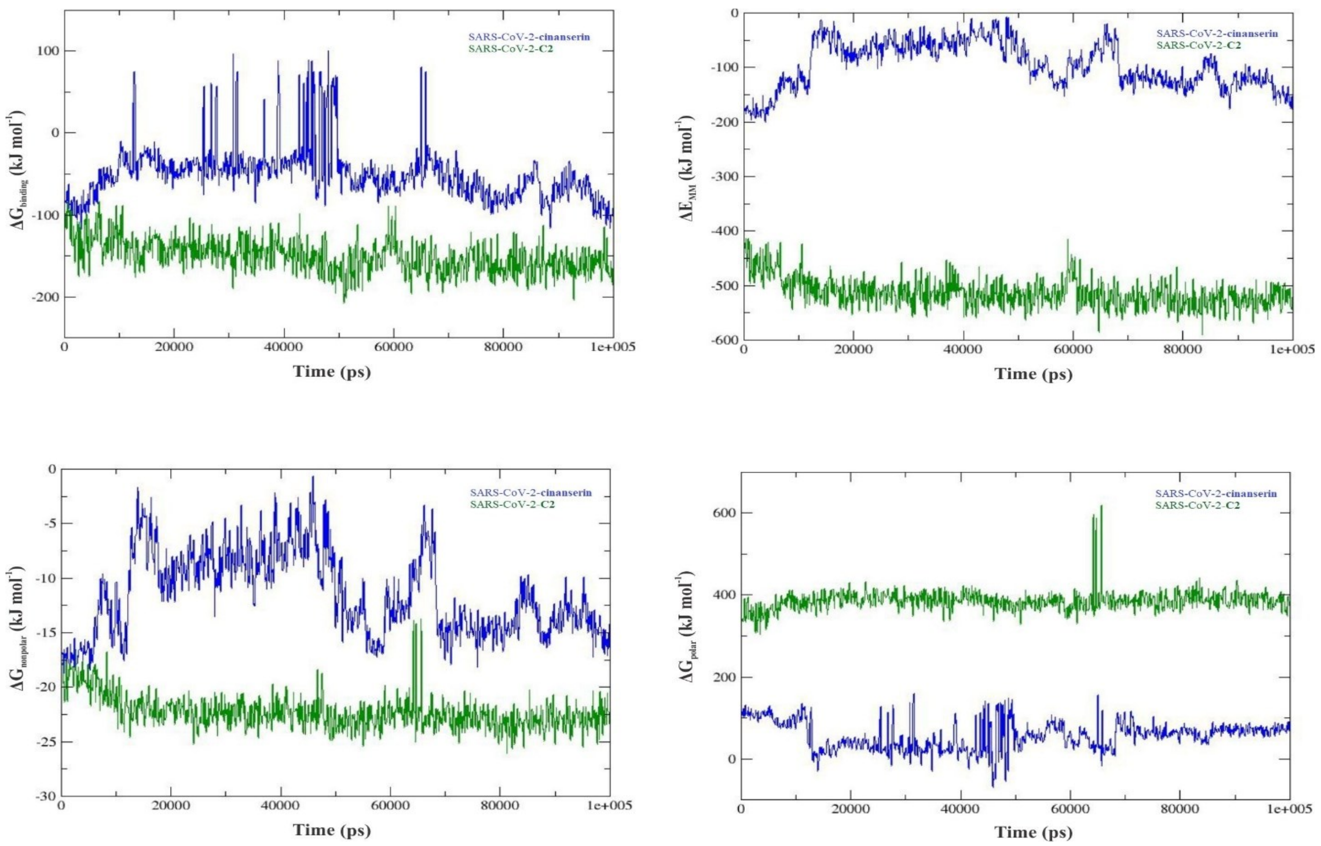


**Figure S7.** Important thermodynamic parameters during 100 ns MD simulation.

**Table S3.** Toxicity of investigated compounds**.**

| | LD50 (mg/kg) | Toxicity Class | Hepatotoxicity | Carcinogenicity | Immunotoxicity | Mutagenicity | Cytotoxicity |
|---|---|---|---|---|---|---|---|
| | | | Probability | Probability | Probability | Probability | Probability |
| **Chloroquine** | 311 | 4 | 0.90 | 0.66 | 0.99 | 0.94 | 0.93 |
| **Cinanserin** | 480 | 4 | 0.66 | 0.62 | 0.68 | 0.64 | 0.66 |
| **L1** | 1600 | 4 | 0.50 | 0.60 | 0.99 | 0.63 | 0.66 |
| **L2** | 2647 | 5 | 0.55 | 0.54 | 0.92 | 0.58 | 0.62 |
| **L3** | 2647 | 5 | 0.55 | 0.54 | 0.92 | 0.58 | 0.62 |
| **C1** | 1210 | 4 | 0.54 | 0.55 | 0.98 | 0.54 | 0.64 |
| **C2** | 3200 | 5 | 0.59 | 0.58 | 0.88 | 0.57 | 0.62 |
| **C3** | 3200 | 5 | 0.59 | 0.58 | 0.90 | 0.57 | 0.62 |

[a]Banerjee P., Eckert O.A., Schrey A.K., Preissner R.: **ProTox-II: a webserver for the prediction of toxicity of chemicals.** Nucleic Acids Res (Web server issue 2018)

Literature


[1] A. Adamu, R.A. Wahab, M.S. Shamsir, F. Aliyu, F. Huyop, Deciphering the catalytic amino acid residues of L-2-haloacid dehalogenase (DehL) from Rhizobium sp. RC1: An in silico analysis, Comput. Biol. Chem. 70 (2017) 125–132. https://doi.org/10.1016/j.compbiolchem.2017.08.007.

[2] A. Singh, M. Das, A. Grover, Molecular mechanism of acetoacetyl-CoA enhanced kinetics for increased bioplastic production from Cupriavidus necator 428, J. Biomol. Struct. Dyn. 38 (2020) 827–840. https://doi.org/10.1080/07391102.2019.1590239.
